# Spatiotemporal analysis of indigenous and imported dengue fever cases in Guangdong province, China

**DOI:** 10.1186/1471-2334-12-132

**Published:** 2012-06-12

**Authors:** Zhongjie Li, Wenwu Yin, Archie Clements, Gail Williams, Shengjie Lai, Hang Zhou, Dan Zhao, Yansha Guo, Yonghui Zhang, Jinfeng Wang, Wenbiao Hu, Weizhong Yang

**Affiliations:** 1 Key Laboratory of Surveillance and Early-warning on Infectious Disease, Chinese Center for Disease Control and Prevention, Beijing, 102206, China; 2 School of Population Health, The University of Queensland, Brisbane, 4006, QLD, Australia; 3 LREIS, Institute of Geographic Sciences and Natural Resources Research, Chinese Academy of Sciences, Beijing, 100101, China; 4 Guangdong Province Center for Disease Control and Prevention, Guangzhou, Guangdong province, 510300, China

## Abstract

**Background:**

Dengue fever has been a major public health concern in China since it re-emerged in Guangdong province in 1978. This study aimed to explore spatiotemporal characteristics of dengue fever cases for both indigenous and imported cases during recent years in Guangdong province, so as to identify high-risk areas of the province and thereby help plan resource allocation for dengue interventions.

**Methods:**

Notifiable cases of dengue fever were collected from all 123 counties of Guangdong province from 2005 to 2010. Descriptive temporal and spatial analysis were conducted, including plotting of seasonal distribution of cases, and creating choropleth maps of cumulative incidence by county. The space-time scan statistic was used to determine space-time clusters of dengue fever cases at the county level, and a geographical information system was used to visualize the location of the clusters. Analysis were stratified by imported and indigenous origin.

**Results:**

1658 dengue fever cases were recorded in Guangdong province during the study period, including 94 imported cases and 1564 indigenous cases. Both imported and indigenous cases occurred more frequently in autumn. The areas affected by the indigenous and imported cases presented a geographically expanding trend over the study period. The results showed that the most likely cluster of imported cases (relative risk = 7.52, p < 0.001) and indigenous cases (relative risk = 153.56, p < 0.001) occurred in the Pearl River Delta Area; while a secondary cluster of indigenous cases occurred in one district of the Chao Shan Area (relative risk = 471.25, p < 0.001).

**Conclusions:**

This study demonstrated that the geographic range of imported and indigenous dengue fever cases has expanded over recent years, and cases were significantly clustered in two heavily urbanised areas of Guangdong province. This provides the foundation for further investigation of risk factors and interventions in these high-risk areas.

## Background

Dengue fever is an acute infectious disease caused by dengue viruses and transmitted by Aedes mosquitoes. The incidence of dengue has grown dramatically around the world in recent decades. The disease is now endemic in more than 100 countries, with about 50 million dengue infections worldwide every year and approximately 2.5 billion people living in dengue-endemic countries. The South-east Asian and Western Pacific regions have the highest burden [1].

In 1978, dengue fever re-emerged in mainland China, in Foshan City of Guangdong province, after disappearing for about 30 years [2]. Since then, outbreaks and epidemics of dengue fever, with cases of dengue hemorrhagic fever (DHF), were reported sequentially in Hainan, Guangxi, Fujian, Zhejiang and Yunnan provinces. All of these provinces are located in the south-east coastal regions or national border with the countries of South-east Asia [3,4]. From 1978 to 2008, a total of more than 650,000 cases were documented in mainland China, resulting in hundreds of deaths [4]. Up to now, dengue fever in mainland China is still characterized as an imported disease, without recognized evidence of its endemic status [5]. Imported dengue cases are regarded as playing a key role in initiating outbreaks in China [4,6,7].

During recent years, with the rapid development of geographic information systems (GIS), methods of spatial analysis have been increasingly applied to infectious diseases, especially zoonoses and vector-borne diseases [8-12]. Identification of spatial high-risk areas can help guide local health departments to formulate public health strategies, initiate early preventive measures and conduct enhanced surveillance, thereby reducing the risk of epidemics occurring [13-15]. In this study, we aim to explore the spatiotemporal characteristics of both indigenous and imported dengue fever cases during recent years in Guangdong province and to detect space-time clusters of cases, so as to identify high-risk areas in the province and thereby help plan resource allocation for dengue prevention and intervention.

## Methods

### Study area

**Figure 1:**
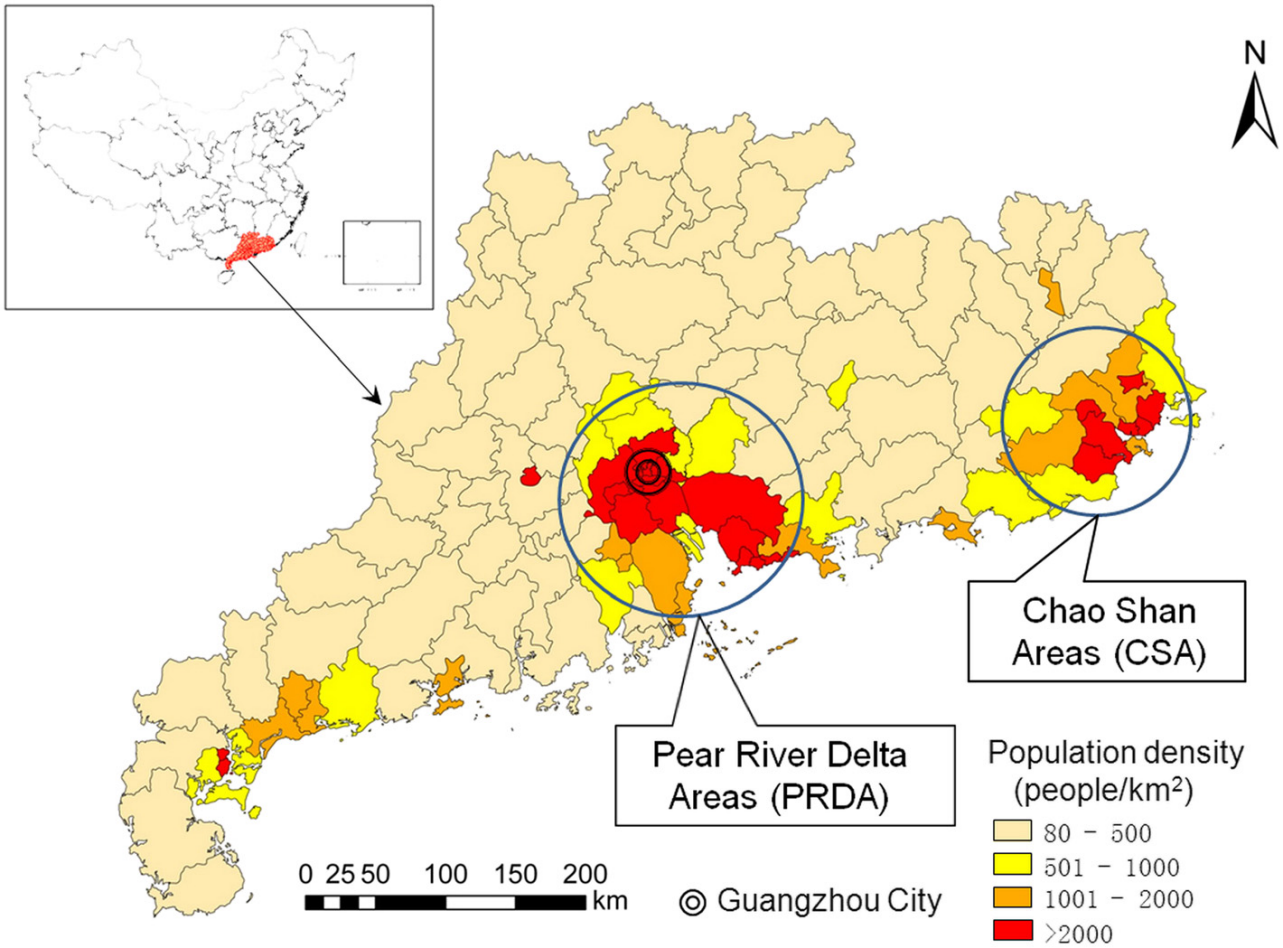
The population density at the county level, Guangdong province, China, 2010.

Guangdong is the province that has had the highest incidence of dengue fever in mainland China during the last decade, accounting for more than 80% of all cases [16]. It lies on the south-east coast of mainland China, covering about 180,000 km^2^, with Guangzhou city as the provincial capital. It is warm and moisture all the year round with average temperatures ranging from 19 to 26 °C, and with a rainy season from April to September [17]. The population reached about 104 million people in 2010, having increased by more than 17.8 million people compared to 2000 [18]. There are greater than 104 million visits by international travellers and 395 million visits by domestic travellers per year [17]. The province includes 121 counties and 2 special administrative regions with no county sub-divisions. In this study, all these 123 areas were regarded as ‘counties’ so as to conduct the spatial analysis at a standardized administrative level across the whole province. The mean population per county is about 783,000 people (ranging from 78,800 to 7.1 million). The population density by county ranges from 83 to 20,945 people/km^2^, with the population being concentrated in the Pearl River Delta Area (PRDA) and the Chao Shan Area (CSA) (Figure 1).

### Data collection

In this study, we obtained data on dengue fever cases in Guangdong from 2005 to 2010, from the Chinese Center for Disease Control and Prevention (China CDC). In China, Dengue fever is a notifiable disease, and all cases of dengue fever were diagnosed according to the unified diagnostic criteria issued by Chinese Ministry of Health, which includes the definitions of clinically diagnosed and laboratory confirmed cases [19]. Clinically diagnosed cases were identified by local experienced physicians according to the clinical manifestations and epidemiologic exposure history. Laboratory confirmed cases were determined basing on clinically diagnosed cases presenting with any of the following lab test results relating to dengue: a 4-fold increase in specific IgG antibody titre, positive on a PCR test or virus isolation and identification test [19]. Both clinically diagnosed and laboratory confirmed cases were included in this study.

A standard form was adopted by local physicians and epidemiologist to collect individual information on each dengue fever case, including age, address, date of onset, diagnosis, laboratory test result and travel history. Routine case reporting is done by hospitals. However, because dengue fever is an emerging disease in China, possible dengue fever cases are also traced by active field investigation when outbreaks occur in the community. Diagnosis in this case involves laboratory testing by the local public health institute. Thus, dengue surveillance involves both passive and active case detection. All dengue fever cases were distinguished as indigenous or imported cases according to the corresponding case definition. An imported case of dengue fever is defined as the case that came from dengue-affected areas outside of mainland China with a history of being bitten by mosquito within 15 days before the onset of illness and has no history of being bitten by mosquitoes in domestic regions, or the gene sequence of the virus isolated from the case is highly homologous with that reported by the country to/from which the patient had travelled. Indigenous cases are defined according to the absence of evidence for the case being imported.

Population data for every county in Guangdong from 2005 to 2010 were retrieved from the National Bureau of Statistics of China. Latitude and longitude coordinates of the centroids of each county in Guangdong were obtained from the Chinese Institute of Geographic Sciences and Natural Resources Research.

### Geographic distribution of disease incidence and space-time clusters

The annual dengue fever incidence was calculated by dividing the number of annual cases by the corresponding population, and multiplied by 100,000. The cumulative incidence by county was mapped to present the geographic distribution of the occurrence of dengue fever cases, which equaled the cumulative new dengue fever cases between 2005–2010, divided by the whole population in the corresponding county in 2005, multiplied by 100,000. To assess changing spatial and temporal patterns of newly affected counties from 2005 to 2010, we plotted the newly affected and previously affected counties by year. We defined counties with dengue fever cases occurring for the first time since 2005 as being newly affected in the corresponding year. These counties were then regarded as existing affected areas in the following years.

The space-time scan statistic was calculated on the imported and indigenous dengue fever cases to test whether the cases were distributed randomly over space and time and, if not, to locate space-time clusters and determine their statistical significance (inference which cannot be made from simple visualisation of the raw data). The software SaTScan version 9.0 was employed to conduct a retrospective space and time scan statistic test [20,21]. In this study, cases were assumed to be Poisson-distributed in each location. The program applies a likelihood function to scanning windows, which move over areas and centre on the centroid of each county. The statistic compares observed and expected case numbers inside and outside the scan window to detect clusters that are least likely to have occurred by chance. The statistical significance of each cluster is obtained through Monte Carlo hypothesis testing, with the number of Monte Carlo replications set to 999. The relative risk (RR) is given by the SaTScan software to present the risk of disease within the scanning window compared to that outside of the scanning window. The window with the maximum likelihood is the most likely cluster, that is, the cluster least likely to be due to chance, and a p-value is assigned to this cluster. SaTScan also identifies secondary clusters in the data set in addition to the most likely cluster, and orders them according to their likelihood ratio test statistic. In this study, only the most likely cluster and the secondary clusters are reported if the p-value of the clusters is below 0.05. In our study, the spatial unit was the county of the case’s residence and the temporal unit was the month of the case’s onset of illness, which were obtained from the individual information of the recorded cases. In the SaTScan software, a default maximum spatial cluster size of 50% of the whole population was used, so as to detect the large clusters that tend to have a small relative risk but a high statistical significance. Furthermore, a maximum spatial cluster size of 10% of the whole population was further employed to detect possible subclusters with smaller size. The maximum temporal cluster size was 50% of the study period.

The GIS software ArcView version 9 (ESRI, Redlands, CA, USA) was used to visualize the population density, cumulative incidence, newly and existing affected areas, and dengue fever clusters.

## Results

### Descriptive analysis

1658 dengue fever cases were reported in Guangdong province from 2005 to 2010, with 94 (5.7%) imported cases and 1564 (94.3%) indigenous cases. 87 (92.56%) imported cases and 1280 (81.84%) indigenous cases were laboratory confirmed. Annual variation in the size of epidemics was striking, with the highest peak occurring in 2006 (1010 cases) and the smallest in 2005 (only 6 cases) (Table 1). Imported cases occurred every year, and there were no indigenous cases recorded in 2005. A total of 35 counties (28.5%) were affected by dengue fever, with about 40 million residents in those counties, accounting for about 45% of the total population of Guangdong province. Among them, 28 (22.8%) counties had imported cases, and 23 (18.7%) counties had indigenous cases. Furthermore, 1 county continuously had dengue fever in all of the six years from 2005 to 2010, and 2 counties had cases in 5 of those years. All these 3 counties were located in the PRDA. Additionally, 12 counties had dengue cases in 3–4 years and 20 counties in 1–2 years over the 6-year study period.

**Table 1:** Descriptive statistics of dengue fever in Guangdong province, China, 2005–2010

**Indicators**	**2005**	**2006**	**2007**	**2008**	**2009**	**2010**	**Total**
All cases	6	1010	397	87	19	139	1658
Imported cases (% of all cases)	6(100)	3 (0.3)	22(5.5)	21(24.1)	15(78.9)	27(19.4)	94 (5.7)
Indigenous cases (% of all cases)	0 (0)	1007(99.7)	375(94.5)	66(75.9)	4 (21.1)	112(80.6)	1564(94.3)
Incidence rate (1/100,000)	0.01	1.10	0.43	0.09	0.02	0.14	1.82*
No. county with cases (% of all counties)	4(3.3)	16 (13.0)	20 (16.3)	13(10.6)	13(10.6)	20 (16.3)	35 (28.5) **†**
No. county with imported cases (% of all counties)	4(3.3)	2 (1.6)	16 (13.0)	9 (7.3)	11 (8.9)	14 (11.4)	28 (22.8) **†**
No. county with indigenous cases (% of all counties)	0 (0)	15 (12.2)	9 (7.3)	7 (5.7)	2 (1.6)	12 (9.6)	23 (18.7) **†**

*: the cumulative incidence from 2005 to 2010.

†: the county with cases in more than one year were counted as only once.

**Figure 2:**
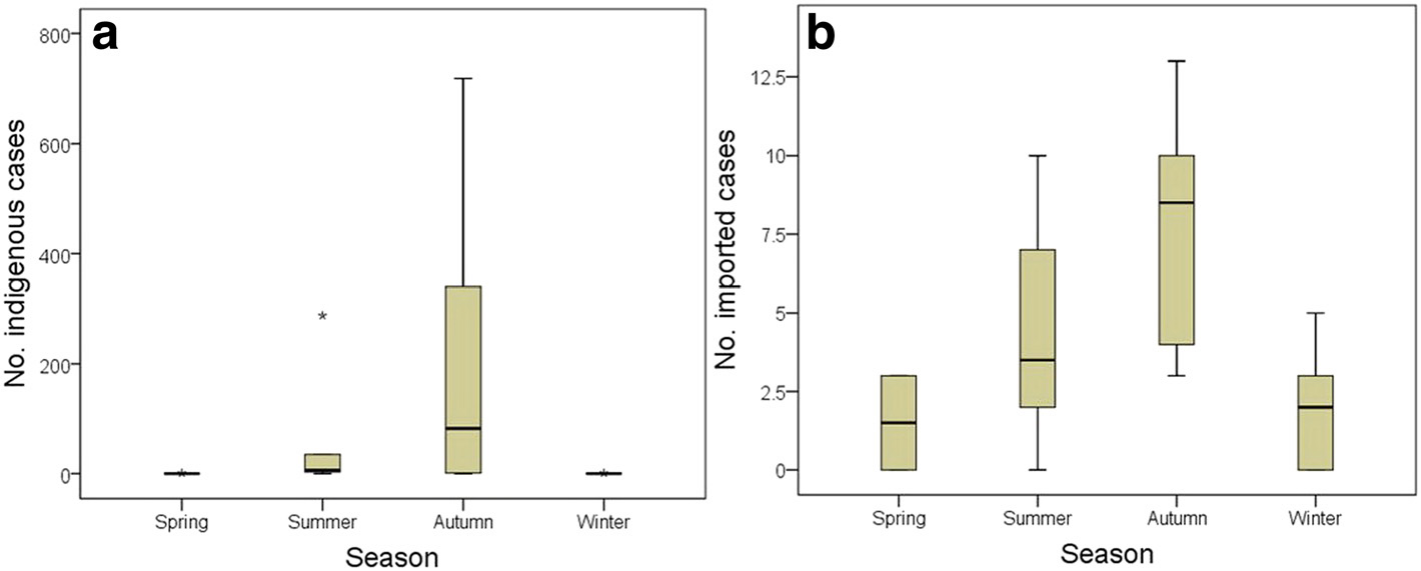
**The seasonal distribution of indigenous and imported dengue fever cases in Guangdong province, China, 2005–2010.a.** Seasonal distribution of indigenous cases; **b.** Seasonal distribution of imported cases. The Boxplots display the values of the 25th, 50th, 75th percentiles. The whiskers extend to the most extreme data point < 1.5 times the inter-quartile range.

The occurrence of both indigenous and imported cases showed definite seasonal characteristics. Cases were relatively rare in winter and spring, with a slightly higher number in summer, and a peak in the autumn (Figure 2).

**Figure 3:**
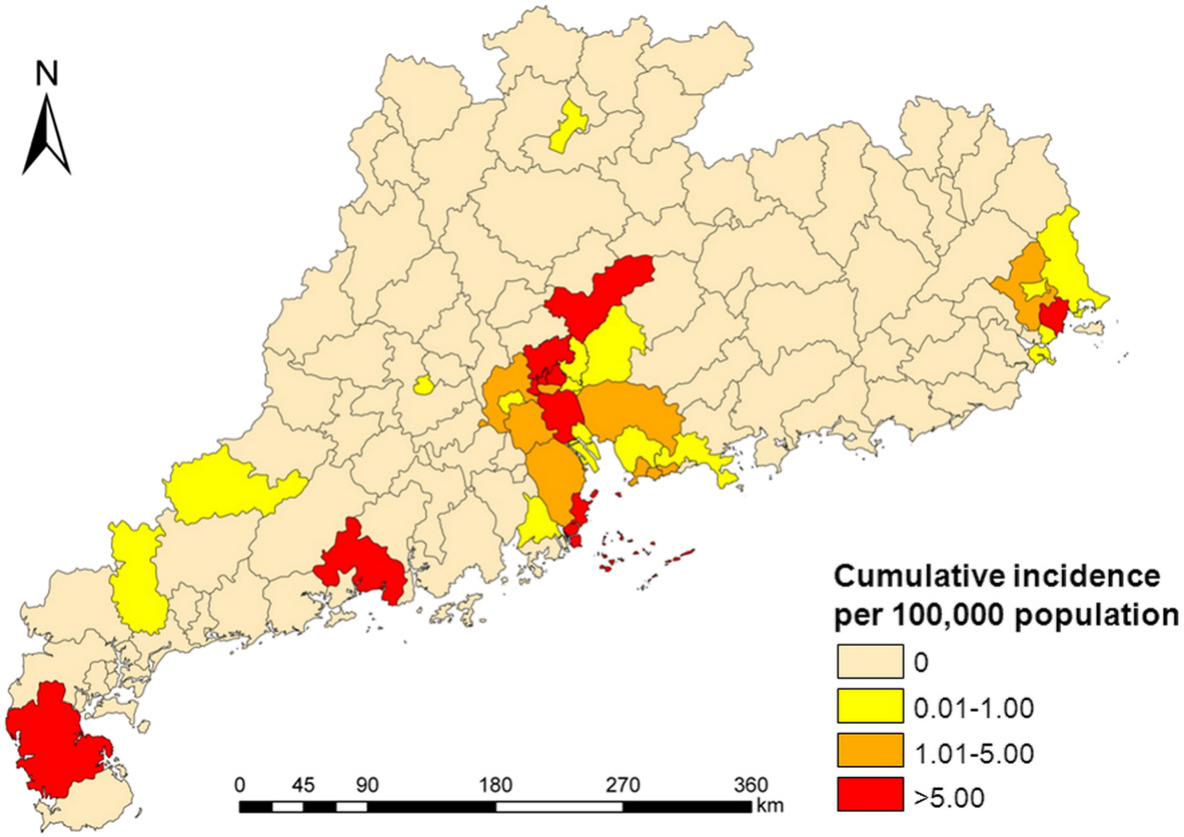
The geographic distribution of cumulative incidence by county in Guangdong province, China, 2005–2010.

**Figure 4:**
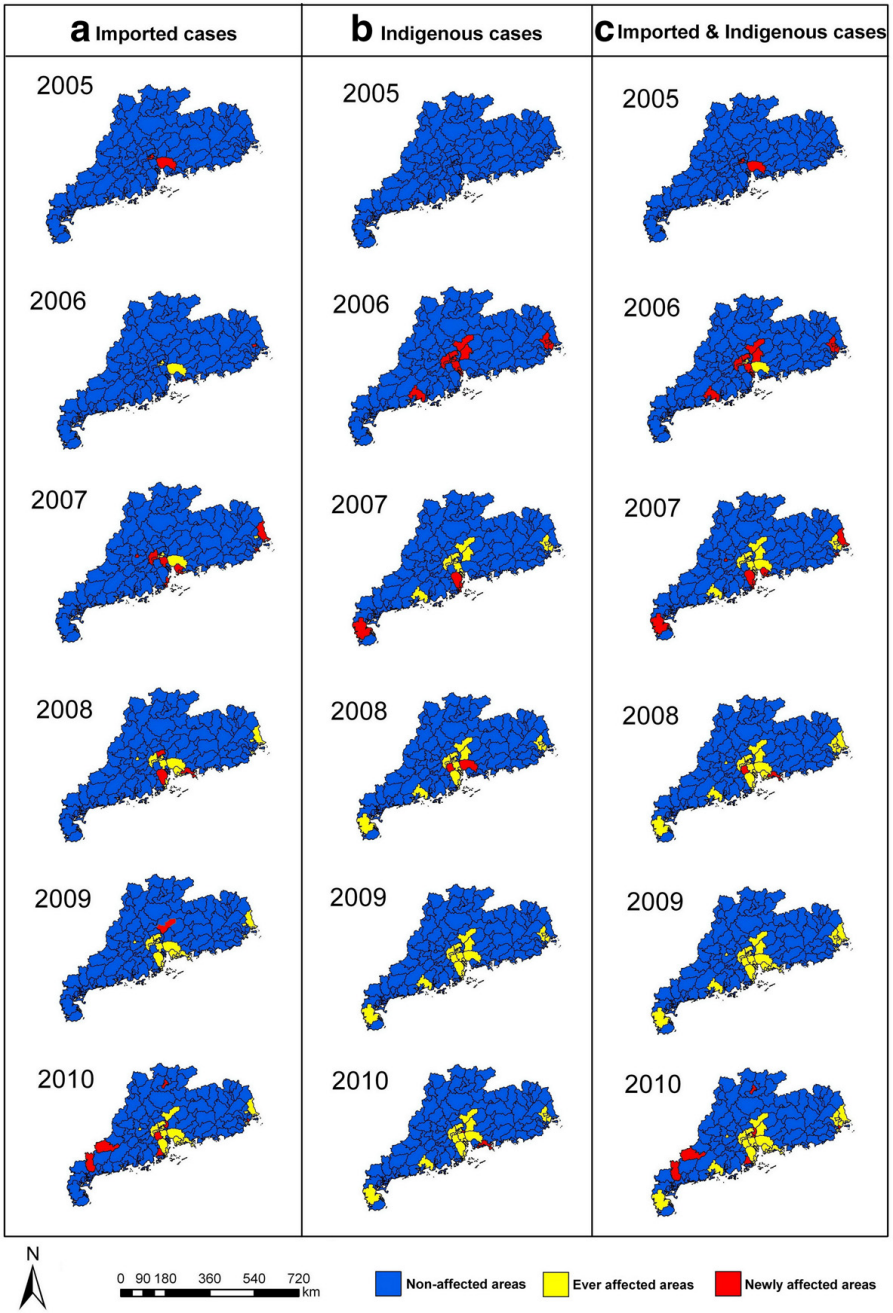
The geographic changing on the affected counties by the imported and indigenous dengue cases in Guangdong province, China, 2005–2010.

The cumulative incidence by county ranged from 0 to 70.6/100,000. There were 10 counties with a cumulative incidence above 5/100,000, which were located in the PRDA (7 counties), CSA (1 county), Yangjiang prefecture (1 county), and Leizhou Peninsula (1 county) (Figure 3). Figure 4 shows that, during the period 2005–2010, the PRDA was the area most affected by both imported and indigenous cases of dengue. The cumulative number of newly affected counties with imported cases increased sharply by year, from 4 counties in 2005 to 28 counties in 2010. Similarly, the cumulative amount of newly affected county with indigenous cases also greatly increased from 0 in 2005 to 23 in 2010. These newly affected counties were mainly located in the PRDA and CSA, with several other counties in the north and west areas of Guangdong province being affected by dengue fever sporadically.

### Spatiotemporal cluster analysis

**Figure 5:**
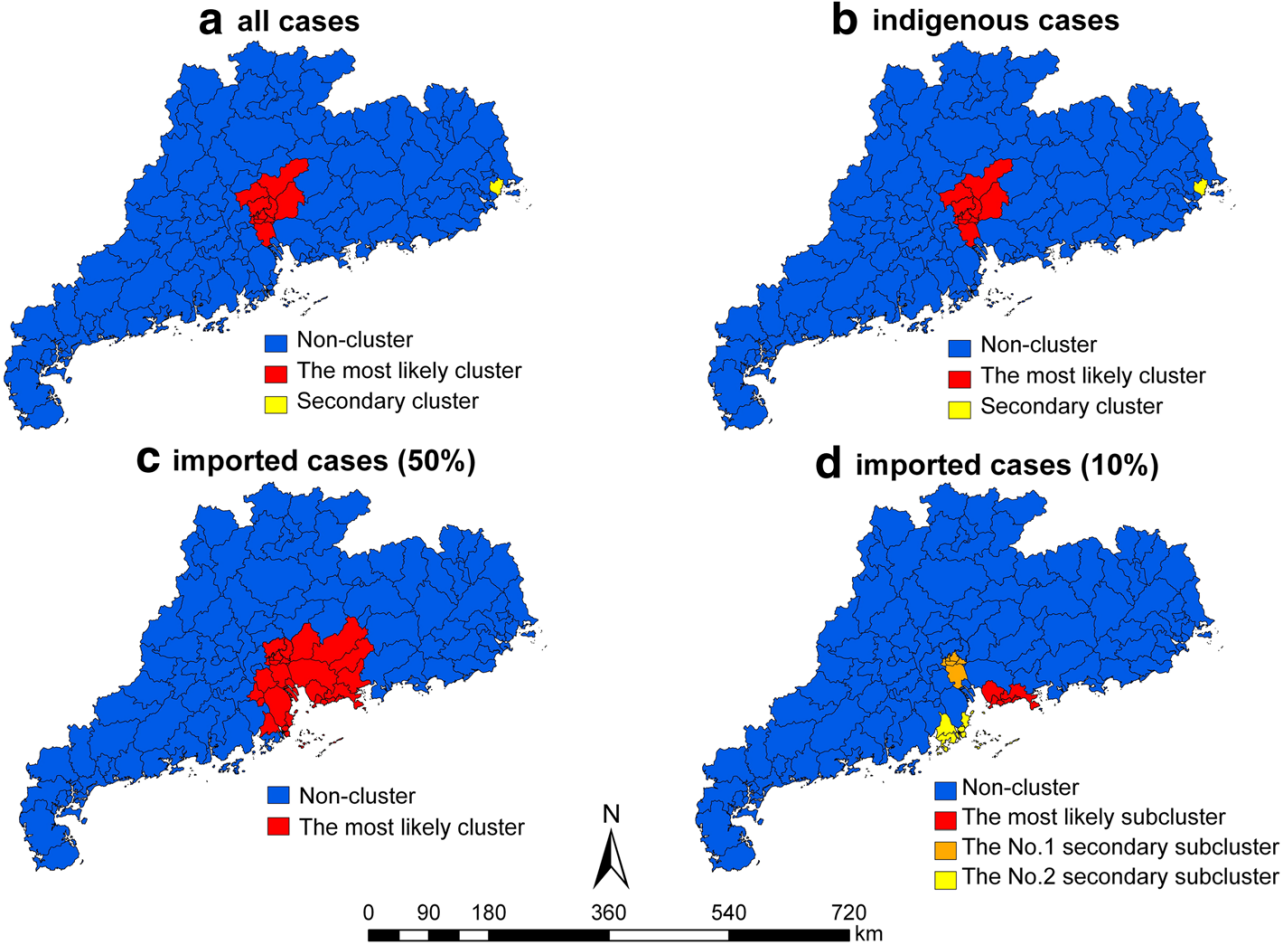
**The clusters of dengue fever by all, indigenous and imported cases in Guangdong province, 2005–2010.a.** Two identified clusters of imported and indigenous dengue fever cases (maximum spatial cluster size ≤ 50% total population); **b.** Two identified clusters of indigenous dengue fever cases (maximum cluster spatial size ≤ 50% total population); **c.** One identified cluster of imported dengue fever cases (maximum cluster spatial size ≤ 50% total population); **d.** Three identified subclusters of imported dengue fever cases (maximum cluster spatial size ≤ 10% total population).

Figure 5 and Table 2 show the results of the spatiotemporal cluster analysis, stratified by all cases, indigenous cases and imported cases. Using a maximum spatial cluster size of ≤50% of the total population, 2 statistically significant clusters were identified for all cases. The most likely cluster covered 11 counties in the PRDA (radius = 50.43 km) with the duration from August, 2006 to November, 2006, having an overall RR of 135.18 (p < 0.001). The secondary cluster included only one district (Chenghai district), located in the CSA with the duration from September, 2006 to October, 2006. This cluster had 177 cases with a RR of 429.69 (p < 0.001). Using the same maximum cluster size, but only looking at indigenous cases, the clusters found were identical to the analysis for all cases. Only one statistically significant cluster of imported cases was detected, which included 26 counties in the PRDA with the duration from April, 2008 to December, 2010. 56 cases were recorded in this cluster, with a RR of 7.52 (p < 0.001).

**Table 2:** Clusters of dengue fever by all, indigenous and imported cases in Guangdong province, China, 2005-2010

**Type of cluster**	**No. counties in the cluster**	**Radius (km)**	**Duration**	**Population**	**Relative risk**	**Log likelihood ratio**	**P-value**
With a maximum spatial cluster size of ≤50% of total population							
1. all cases							
M*	11	50.43	2006/8-2006/11	10,394,229	135.18	2688.65	<0.001
S**†**	1	0	2006/9-2006/10	862,662	429.69	886.44	<0.001
2. indigenous cases							
M*	11	50.43	2006/8-2006/11	10,394,229	153.56	2760.44	<0.001
S**†**	1	0	2006/9-2006/10	862,662	471.25	902.08	<0.001
3. imported cases							
M*	26	89.80	2008/4-2010/12	32,795,835	7.52	44.66	<0.001
With a maximum spatial cluster size of ≤10% of total population							
1. all cases							
same to that with a maximum spatial cluster size of ≤50% of total population							
2. indigenous cases							
same to that with a maximum spatial cluster size of ≤50% of total population							
3. imported cases							
M*	6	27.00	2008/4-2010/10	7,608,517	10.95	36.19	<0.001
S**†**	6	19.47	2007/4-2007/11	6,013,893	13.81	13.27	0.02
S**†**	3	39.40	2007/10	1,342,186	162.28	12.24	0.03

*M: the most likely cluster.

† S: the secondary cluster.

Using a maximum spatial cluster size of ≤10% of total population, the clusters for all cases and indigenous cases were identical to the analysis using a maximum spatial cluster size of ≤50% of total population. Furthermore, three subclusters of imported cases were identified. The most likely subcluster covered 6 counties with the duration from April, 2008 to October, 2010, all in Shenzhen city, with RR of 10.95 (p < 0.001); the next secondary subcluster also covered 6 counties, all in Guangzhou city, with the duration from April, 2007 to November, 2007 and RR of 13.81 (p < 0.05); and a final second secondary subcluster covered 3 counties, all in Zhuhai city, with the duration of October, 2007 and RR of 162.28 (p < 0.05).

## Discussion

In this study, two high-risk areas for dengue fever were identified in Guangdong province. The PRDA was a high-risk area both for indigenous and imported cases, and one district of the CSA was a high-risk area for indigenous cases. The geographic areas affected by imported and indigenous dengue fever cases expanded across the province from 2005 to 2010.

The two most important mosquito species for transmission of dengue viruses, *Aedes aegypti* and *Aedes albopictus*, have became established in Guangdong province for a long time. The PRDA and CSA are the areas with the highest density of *Ae. albopictus* in the province, having a favourable natural climate for mosquito breeding [3,4,22]. Traditionally, residents in these areas have had the habit of planting flowers in flowerpots in household courtyards, which provides many containers with water around the house, benefitting mosquito breeding. The PRDA is located in the middle of Guangdong province. It is the region with the most rapid economic growth, and has one of the highest population densities in China. As reported by the census in 2010, a mobile population of more than 30 million people came from inland areas of China to live temporarily in Guangdong province, to seek job opportunities. The mobile population is extremely large, accounting for about 1/3 of the total provincial population. It is concentrated in the metropolises especially the cities of Guangdong, Shenzhen and Zhuhai [17]. The mobile population may increase the susceptible population in this region because they originate from inland areas where dengue is absent [4]. The rapid development of urbanization and construction in this region may have also contributed to the transmission of dengue. One outbreak with 35 cases was reported in a subway construction site, among workers from inland areas [23]. All these factors may explain the relative higher risk of indigenous dengue fever cases in PRDA found in this study. Increasing overseas commercial investment, export of labour and foreign tourism have led to more international exchanges in this region, increasing the risk of imported dengue fever cases from endemic areas. Most of the imported cases in Guangdong province came from Southeast Asian countries with endemic dengue fever, including Singapore, Indonesia, Cambodia and the Philippines [4,24]. Several studies have also reported that dengue fever cases from South-east Asian countries have triggered local outbreaks in Guangdong province [3,4,6,24]. The seasonal pattern of epidemics in these Southeast Asian countries and the seasonality of travel to Guangdong were the possible reasons for imported cases presenting a seasonal pattern [24]. Certainly, the relationships between indigenous and imported cases in Guangdong province need to be further investigated in future research.

Our study not only firstly identified clusters of imported dengue fever cases in Guangdong, but also further detected subclusters of imported cases in Guangzhou, Shenzhen and Zhuhai cities. All of these cities are relatively developed areas in the PRDA. With the high volume of frequent population movement in the PRDA, this region is critical to the prevention of dengue both in Guangdong and the whole of China. Enhanced measures should be taken to reduce local transmission of dengue and prevent it spreading to other areas within and outside the province. Another cluster area identified by this study was in the CSA. This has been long known to be a region at risk of imported dengue fever cases because it is the homeland of a large overseas Chinese population in South-east Asia, who are frequent visitors to this region [25]. Our study newly identified one county of CSA as an area with a higher risk of indigenous cases, which may be an indicator of potential changes on the dengue transmission patterns in this region.

Our findings also indicate that affected areas appear to have been expanding in Guangdong over recent years, which indicates potentially increasing risk for unaffected areas in other parts of Guangdong province. These risks are underpinned by domestic and international travel and a global expansion of dengue fever [26-28]. It is interesting that the Leizhou peninsula, where a large epidemic occurred in the 1980s, with about 25,000 cases, was not identified as a cluster area, even though about 200 cases occurred there in September and October of 2007. This requires more investigation and monitoring of the dengue situation in this area.

By contrast to several previous spatiotemporal studies of dengue fever in other countries [15,26,29], our study was stratified by indigenous and imported cases. This provided more specific information for local health departments facing the different risks of imported and indigenous dengue fever cases.

A potential limitation to the study could be underreporting and misreporting of both imported and indigenous cases due to asymptomatic infection, clinical misdiagnosis, and the capacity of some laboratories to test samples. However, because dengue fever is a notifiable disease and a high priority in Guangdong, surveillance data quality supervision was regularly performed in clinics and hospitals. More importantly, the capacity of laboratories to do dengue testing was relatively high in this province. Therefore, we have reasonable confidence in the quality and completeness of reporting.

## Conclusions

In conclusion, we found that both indigenous and imported cases of dengue fever in Guangdong province, the province most affected by dengue fever in China, occurred in spatial and temporal clusters with high-risk areas. The areas newly affected by dengue fever seemed to be expanding across the province. Additionally, the study also demonstrated that travel between endemic and non-endemic areas may influence the observed spatiotemporal distribution of dengue in non-endemic regions. For example, in Guangdong province, imported dengue cases have led to seasonal peaks in dengue risk and spatial clustering in the areas of the province which receive a higher number of travellers from endemic regions in Southeast Asia. These findings could be used to inform risk-based surveillance aimed at identifying high-risk locations and time-periods during which more intensive control measures can be implemented.

## Competing interests

The authors declare that they have no competing interests.

## Authors' contributions

ZJL carried out the whole process of data collation, data analysis and drafted the manuscript. WWY, ZH and YHZ assisted in data review and data interpretation. SJL, ZD, YSG and JFW performed the data collation and data analysis. AC, GW, WBH and YWZ conceived of the study, participated in its design and revised it critically for important intellectual content. All authors red and approved the final manuscript.

## Pre-publication history

The pre-publication history for this paper can be accessed here:

http://www.biomedcentral.com/1471-2334/12/132/prepub
